# Reflectance confocal microscopy features of ink spot lentigo: When in‐vivo digital biopsy can avoid unnecessary excisions

**DOI:** 10.1111/srt.13554

**Published:** 2024-01-04

**Authors:** Federico Venturi, Danela Tassone, Carlotta Baraldi, Aurora Alessandrini, Emi Dika

**Affiliations:** ^1^ Oncologic Dermatology Unit IRCCS Azienda Ospedaliero‐Universitaria di Bologna Bologna Italy; ^2^ Department of Medical and Surgical Sciences (DIMEC) Alma Mater Studiorum University of Bologna Bologna Italy; ^3^ Plastic surgery unit IRCCS Azienda Ospedaliero‐Universitaria di Bologna Bologna Italy

Dear Editor,

Reticulated black solar lentigo, also known as ink‐spot lentigo, is clinically defined by irregular shape and black color resembling an ink‐spot. These lesions are usually located on sun‐exposed areas in the context of multiple solar lentigines and/or seborrheic keratoses.^1^ Differential diagnosis with cutaneous melanoma is mandatory and, in certain cases, difficult at clinical/dermoscopic examination. Herein we present the case of a 72‐year‐old patient with previous history of cutaneous melanoma who was referred to our clinic for an atypical little black spot of the right shoulder which revealed, at reflectance confocal microscopy (RCM), features of an ink‐spot lentigo.

In November 2023, a 72‐year‐old man was sent for consultation at our clinic for a black macule of the right shoulder. Past medical history revealed a pT1a (0.5 mm Breslow thickness) superficial spreading melanoma of his back and a pT1a (0.3 Breslow thickness) superficial spreading melanoma of his right leg excised in 2016 and 2017, respectively. The patient underwent wide local excision with 1 cm tumor margins and subsequent clinical follow up every 6 months for the first 5 years, then once a year, with no evidence of recurrences. At clinical examination we observed a highly pigmented 4 mm macule located on the right shoulder in the context of multiple solar lentigines and seborrheic keratoses. Dermoscopy was not conclusive since it showed a prominent thick black branched pigment network together with dark brown polygonal lines and rhomboidal structures (Figure 1). The lesion developed on sun‐exposed area close to a seborrheic keratosis. We further performed RCM which displayed a ringed pattern with edged papillae defined by small, typical cells demarcating regular papillae, white reticulated collagen and small inflammatory cells within the whole lesion (Figure 2). The diagnosis of an ink‐spot lentigo was confirmed with no need of further excisions. As we know, differential diagnosis with malignancies is mandatory but not that easy when visiting patients with multiple pigmented flat lesions and a previous history of cutaneous melanoma. To date, histopathology represents the gold standard for the diagnosis of most of skin diseases. However, as the years go by, different technologies have been developed and introduced in our daily practice, and RCM emerges as a valuable non‐invasive tool for real‐time visualization of skin lesions.^2^ Its utility extends from the diagnosis of cutaneous neoplasms, delineating cancer margins, monitoring therapeutic interventions, and even aiding in the diagnosis of inflammatory conditions. Particularly, one of its main fields of application is the definition of flat pigmented lesions, as for our patient. In fact, previous studies have demonstrated that RCM is useful for identifying the histological substrate of dermoscopic features in such lesions, aiding to differentiate benign tumors from malignancies.^3^, ^4^, ^5^ In our case, RCM provided to be a useful tool for the correct definition of a benign lesion, avoiding a biopsy and reassuring a patient with a previous diagnosis of cutaneous melanoma thanks to a quick and painless analysis.

**FIGURE 1:**
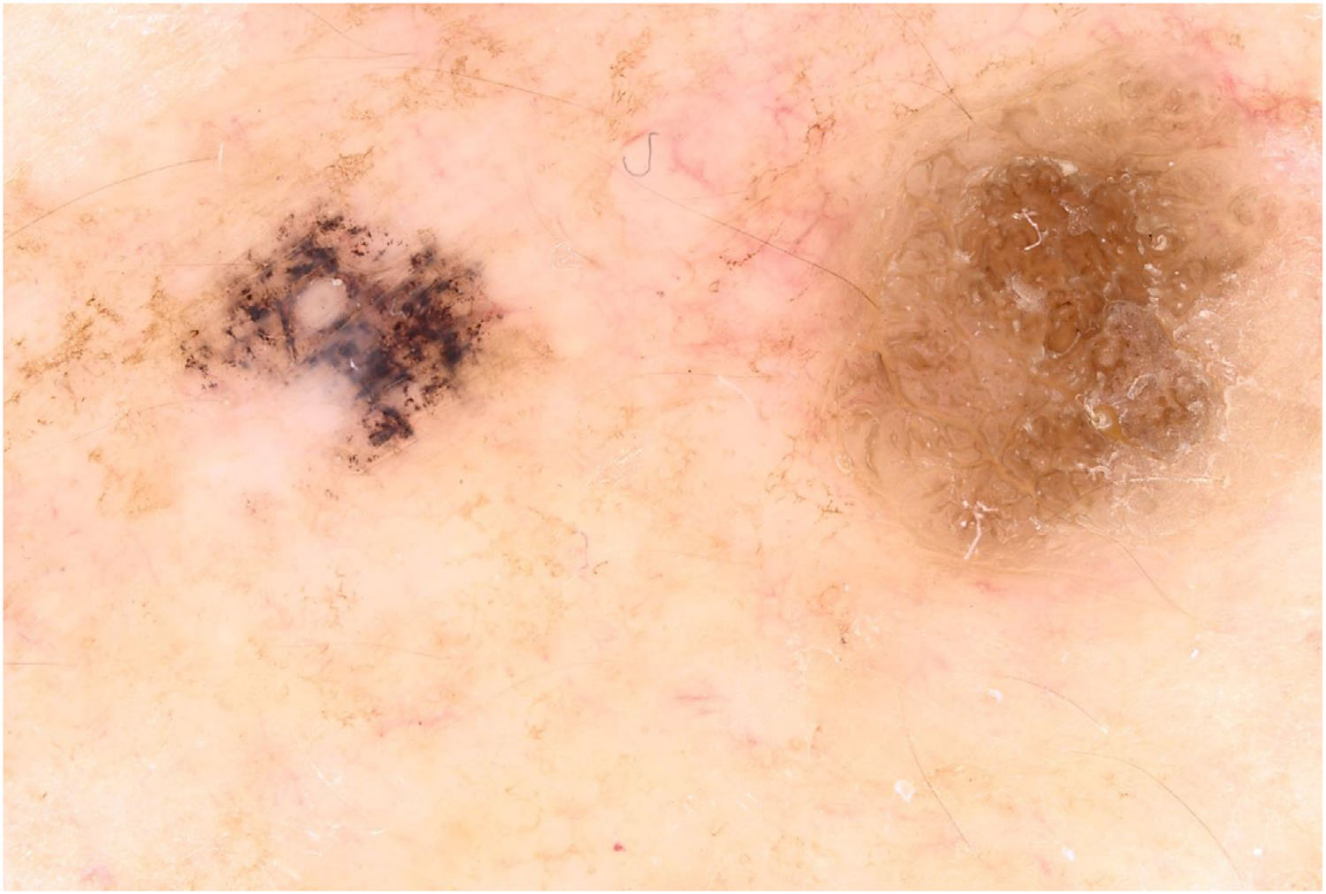
Dermoscopic presentation of an atypical ink‐spot lentigo (on the left) with prominent thick black branched pigment network and dark brown polygonal lines and rhomboidal structures located on the right shoulder of a 72‐year‐old patient at side of a seborrheic keratosis.

**FIGURE 2:**
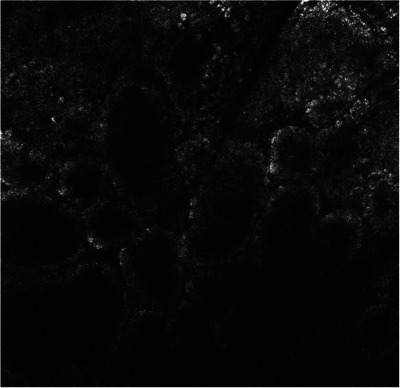
RCM examination of the ink‐spot lentigo showing a ringed pattern with edged papillae defined by small, typical cells demarcating regular papillae, white reticulated collagen and small inflammatory cells. RCM, reflectance confocal microscopy.

## CONFLICT OF INTEREST STATEMENT

The authors have no conflict of interest to declare.

## PATIENT CONSENT

The patient in this manuscript have given written informed consent to publication of his case details.

## Data Availability

Data sharing is not applicable to this article as no new data were created or analyzed in this study.
